# Quadratic relationships between group size and foraging efficiency in a herbivorous primate

**DOI:** 10.1038/s41598-018-35255-0

**Published:** 2018-11-13

**Authors:** Cyril C. Grueter, Andrew M. Robbins, Didier Abavandimwe, Veronica Vecellio, Felix Ndagijimana, Tara S. Stoinski, Martha M. Robbins

**Affiliations:** 1 Max Planck Institute for Evolutionary Anthropology, Leipzig, Germany; 2 The Dian Fossey Gorilla Fund International, Atlanta, USA; 3 School of Human Sciences, The University of Western Australia, Perth, Australia; 4 Centre for Evolutionary Biology, School of Biological Sciences, The University of Western Australia, Perth, Australia; 5 Zoo Atlanta, Atlanta, USA

## Abstract

The effect of feeding competition on foraging efficiency is an important link between ecological factors and the social organization of gregarious species. We examined the effects of group size on daily travel distances, activity budgets, and energy intake of mountain gorillas in Rwanda. We measured daily travel distances of five groups, activity budgets of 79 gorillas in nine groups, and energy intake data for 23 adult females in three groups over a 16-month period. Travel distances and the proportion of time spent traveling increased with size for most groups, which would be expected if their foraging efficiency is limited by intragroup feeding competition. However, travel distances and times decreased for the largest group, which also had higher energy intake rates than intermediate sized groups. The improved foraging efficiency of the largest group may be explained by advantages in intergroup contest competition. The largest group had much lower home range overlap than the other study groups which may be due to groups avoiding one another as a result of male mating competition. Collectively, our results indicate that intermediate sized groups had the lowest foraging efficiency and provide a new twist on the growing evidence of non-linear relationships between group size and foraging efficiency in primates.

## Introduction

Feeding competition has been considered one of the most important links between ecological factors and the social organization of gregarious species^1,2^. Socioecological models predict that ecological factors primarily determine the foraging efficiency, reproductive success, and distribution of females; which in turn will influence the distribution of males^3,4^. The relationships between foraging efficiency and group size can be influenced by both scramble competition and contest competition, which can occur both within groups and between groups^5,6^.

Within-group scramble competition (WGS) is predicted to create a positive correlation between group size versus daily travel times and distances^7,8^. If food is distributed into discrete patches, then large groups may need to visit more patches to obtain the same amount of food per individual^9,10^. When food is more evenly dispersed, individuals in larger groups may have a greater probability of encountering a food site where another group member has already eaten, so they may need to travel farther to find fresh sites^11,12^.

If foraging groups advance through their habitat by “pushing forward” the members in the front, then WGS and/or within-group contest competition (WGC) could also contribute to a positive correlation between group size and travel^9,13,14^. WGC can involve aggressive displacement by dominant individuals, or subordinates may move preemptively to avoid such aggression^15,16^. Such competition is typically associated with rank related differences in foraging efficiency and reproductive success^17,18^. WGC may be more intense when foods are small enough to be monopolized and take a long time to consume^19,20^. We will use the term “intragroup feeding competition” to encompass both WGS and WGC.

Between-group contest competition (BGC) may create a negative correlation between group size and travel^7,8^. Small groups may travel farther to avoid encounters with large groups, and they may be displaced from food patches where such encounters occur^21,22^. BGC is predicted when patches of food are large enough to accommodate an entire group, yet small enough that the group can exclude other rivals^1,23^. For species with sexual dimorphism, the quality and quantity of males may be more important for winning contests between groups than the total number of members in a group, and feeding competition may overlap with male mating competition^24–27^. We will use the term “intergroup contest competition” to refer to BGC while acknowledging a potential influence of mating competition.

The fourth permutation of feeding competition, between-group scramble competition (BGS), is not expected to involve correlations with group size, but may lead to lower foraging efficiency at higher population density^5,28^. Increasing population density can reduce the abundance of food, which may intensify the other types of feeding competition^29^. For all four types of competition, lower foraging efficiency may involve greater depletion of food sites, and individuals may settle for closer sites with lower quality, resulting in lower food intake rates^8,30^. To compensate for lower intake rates, as well as the energetic costs of greater travel, individuals may spend more time feeding^28,31^.

A combination of intragroup feeding competition and BGC has been evoked to explain why optimal foraging efficiency occurs at intermediate group sizes^32,33^. Intragroup feeding competition can reduce the foraging efficiency for large groups, while BGC can reduce the foraging efficiency of small groups. Furthermore, an upper limit for optimal group sizes has been attributed to infanticide, and a lower limit has been attributed to predation risks^1,21,34^. Nonetheless, group sizes can vary considerably both within and among species, and the consequences of those variations have not been well quantified^14^.

Here we investigate the effects of group size on the foraging efficiency of mountain gorillas in the Virunga volcano region. The Virunga mountain gorillas are expected to have weak feeding competition, primarily because their food is abundant^35,36^. Distances between patches are small, which limits the extra travel requirements if larger groups must visit more patches^37,38^. Food sites are numerous and densely distributed within those patches, so extra foraging costs are minimal if some sites have already been depleted, and individuals have little incentive to compete for any particular spot^39–41^. The Virunga mountain gorillas have been considered unlikely candidates for BGC because intergroup encounters have traditionally been infrequent and intergroup aggression is mainly limited to male mating competition^42–44^. Male mating competition may be more influential than BGC, as groups try to retain females by avoiding encounters with potential rivals, and daily travel distances can be longer after encounters occur^35,45–47^. Hypothetically, competition among males could also be based on resource defense, but significant evidence has not been found to support such a possibility for gorillas^48,49^.

Previous studies have supported expectations that group size has little influence on the foraging efficiency of this population. Inconsistent results have emerged from analyses of daily travel distance versus group size^35,45^. Time spent feeding was significantly longer in larger groups, but the increase was only three percentage points across a three-fold variation in group size^39^. Dominance rank did also not have a significant effect on the energy intake rates or the proportion of time spent traveling, and rank-related differences in female reproductive success might be due to female quality rather than contest competition^41,50,51^. Thus, both types of intragroup feeding competition (WGS and WGC) appear to be weak in this species^44^. Subsequent to most of the previous studies of group size, however, some groups have become larger and intergroup encounters have become more frequent, which increases the potential for both intragroup feeding competition and intergroup contest competition^52^.

To examine the effects of group size on foraging efficiency, we looked at the daily travel distances, activity budgets, and energy intake rates of the Virunga mountain gorillas. If intragroup feeding competition is the main influence on foraging efficiency, then we expect larger groups to have longer travel distances and times, as well as lower energy intake rates. We predict the opposite patterns if intergroup contest competition predominates. If both types of competition are determining the optimal group size for this population, then foraging efficiency should be highest at intermediate sizes, with lower values for smaller and larger groups (if the probability of winning is based on group size and male number). We discuss our results within the context of the socioecological theories about foraging efficiency and social organization.

## Results

**Figure 1:**
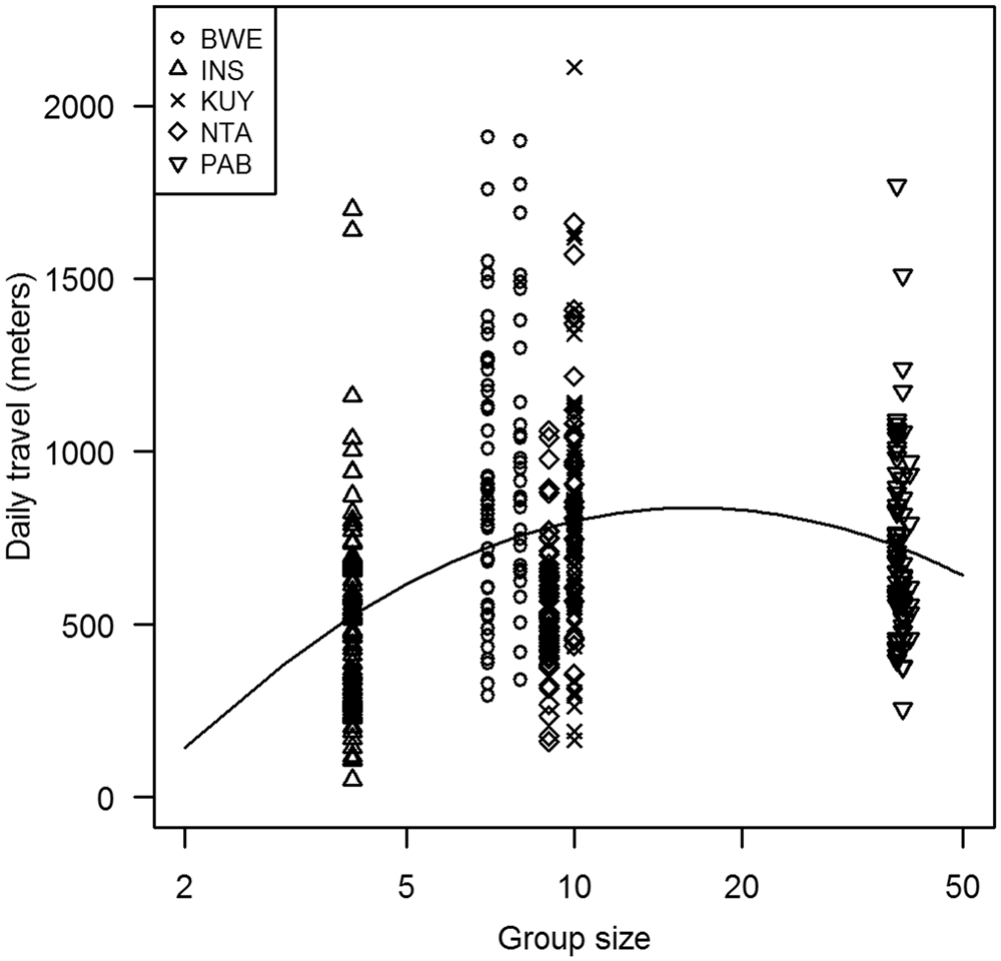
Group size versus the daily travel distance (meters). Each data point represents one day that the group travel was measured. The line is based on a bivariate regression between daily travel versus group size and size-squared. Detailed results from a more complete model are shown in Table 1a.

Daily travel distances averaged 712.5 ± 339.6 meters (n = 448 group-days in five groups). The daily travel distances showed a significant quadratic relationship with group size (Table 1a). The linear mixed model that we used for statistical analysis predicted longer travel distances at intermediate group sizes, however, so the curvature is in the opposite direction of expectations (Fig. 1). Visual inspection suggests that the quadratic term might be excessively sensitive to the data from the largest or smallest group. The p-value remained significant when we removed the largest group from the analysis, however, which suggests that the effects of group size were already leveling off for the other groups. The p-value also remained significant when we removed the smallest group (or any of the other groups). The linear term for group size was also significant in the original model, but not when we removed the quadratic term (p = 0.10).

**Table 1:** Statistical details from the linear mixed models.

Variable	Estimate	StdErr	t-value	p-value
***(a) daily travel distances (meters***)				
Intercept	0.263	0.196	1.344	NA
Group size	0.457	0.170	2.695	0.013
Group size^2^	−0.308	0.131	−2.358	0.030
Rainfall	−0.136	0.040	−3.382	<0.001
Temporal ac	0.281	0.040	7.003	<0.001
**(** ***b*** **)** ***proportion of time spent traveling***				
Intercept	0.542	0.091	5.931	NA
Offset	0.193	0.012	16.333	0.000
Group size	−0.054	0.072	−0.752	0.348
Group size^2^	0.129	0.052	2.489	0.041
Rainfall	−0.020	0.011	−1.836	0.069
Temporal ac	0.076	0.011	6.829	<0.001
**(** ***c*** **)** ***proportion of time spent feeding***				
Intercept	2.259	0.075	30.240	NA
Offset	0.543	0.022	24.595	<0.001
Group size	−0.098	0.048	−2.036	0.033
Group size^2^	−0.040	0.038	−1.053	0.224
Rainfall	−0.030	0.021	−1.419	0.147
Temporal ac	0.052	0.021	2.474	<0.001
**(** ***d*** **)** ***energy intake rate*** **(** ***kJ per minute*** **)**				
Intercept	2.959	0.053	55.937	NA
Offset	0.204	0.005	42.994	0.000
Size category	−0.114	0.049	−2.354	0.043
Temporal ac	0.101	0.013	7.994	<0.001
**(** ***e*** **)** ***energy intake per food site*** **(** ***kJ*** **)**				
Intercept	3.541	0.052	67.768	NA
Size category	−0.162	0.062	−2.617	0.031
Temporal ac	0.073	0.016	4.609	<0.001
**(** ***f*** **)** ***meters traveled between food sites***				
Intercept	1.066	0.032	33.492	NA
Size category	0.034	0.048	0.714	0.457
Temporal ac	0.105	0.019	5.500	<0.001

Estimate and standard error for the coefficient of each variable. Temporal ac is the control variable for temporal autocorrelation.

**Figure 2:**
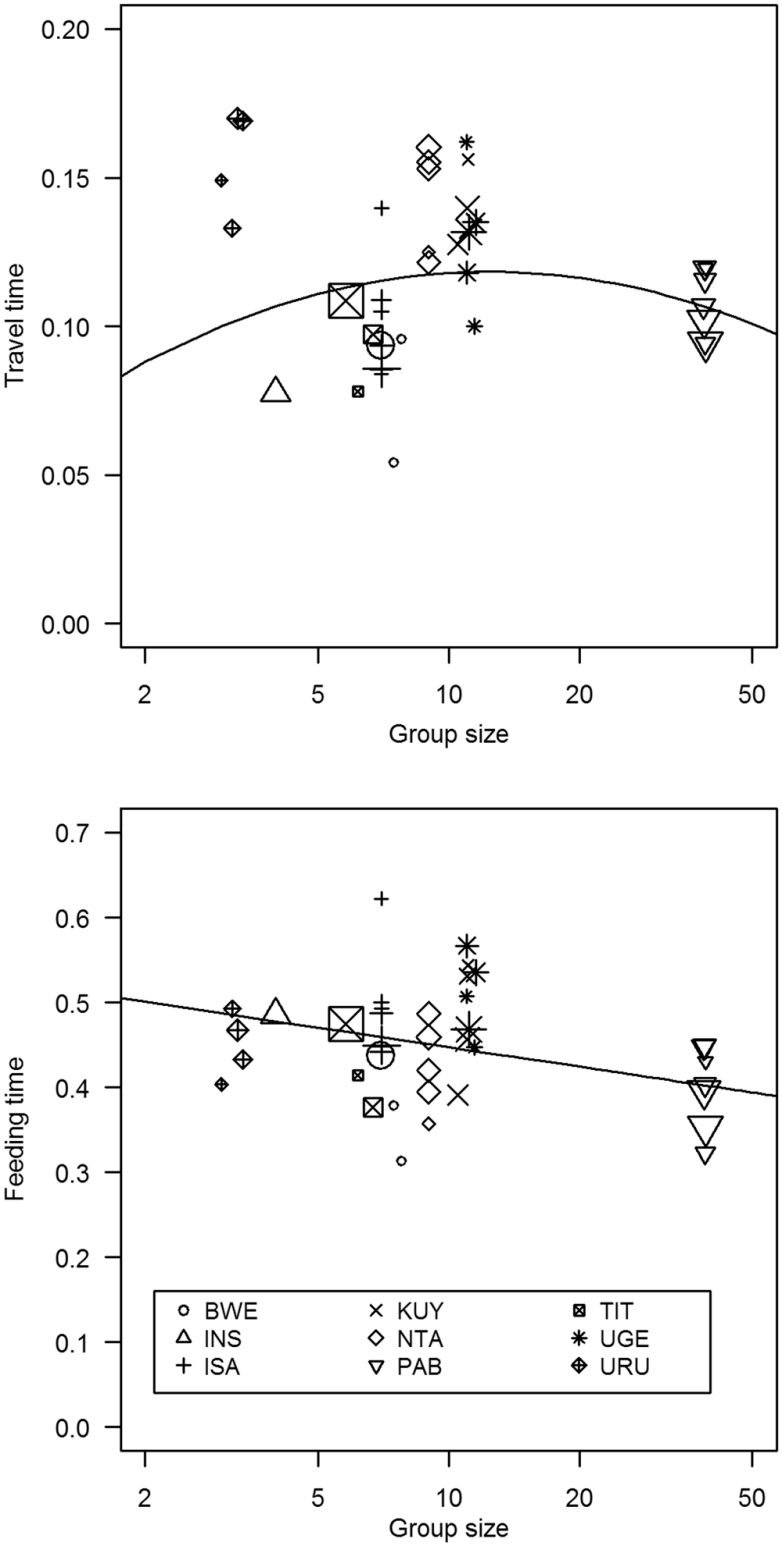
Group size versus the proportion of time that gorillas spent traveling (**a**) or feeding (**b**). For purposes of clarity, the results are aggregated into one data point for each group in each habitat, and data point with less than 100 scans are omitted. The size of each symbol reflects the sample size. The legend at the bottom of (**b**) indicates the group ID for each data point.

Using a larger dataset that included four more groups and 1000 more group-days, the results for the proportion of time spent traveling were similar to the daily travel distances (Fig. 2a). The quadratic term for group size was significant, and the model again predicted greater travel for intermediate group sizes (Table 1b). The observed pattern remained significant when we removed the smallest group, but not when we removed the largest group.

The gorillas spent an average of 45.4% ± 3.2% of their time feeding (Table 2). The proportion of time spent feeding showed a significant negative correlation with group size, and the quadratic term was not significant (Table 1c, Fig. 2b). The linear term remained significant if we removed group BWE, KUY, or UGE; it became a statistical trend (0.05 < p < 0.10) if we removed group INS, ISA, NTA, TIT, or URU; and the p-value increased to 0.57 if we removed the largest group.

**Table 2:** Summary of the study groups.

Group	Average group size	Group size range	Daily travel meters	Time spent traveling	Time spent feeding	Energy Intake kJ/min	Energy intake kJ per site	Meters between sites
URU	3.2	(3–5)	—	16.5%	46.5%	—	—	—
INS	4.0	(4–4)	493.6 ± 289.8	8.0%	47.7%	—	—	—
TIT	5.9	(5–7)	—	10.7%	46.2%	—	—	—
ISA	7.0	(7–7)	—	9.2%	46.4%	—	—	—
BWE	7.0	(6–8)	939.7 ± 382.1	9.3%	42.6%	81.4 ± 64.0	167.4 ± 169.5	5.1 ± 7.8
NTA	9.0	(9–9)	651.8 ± 302.0	14.9%	44.3%	82.3 ± 63.7	192.7 ± 198.4	4.5 ± 4.6
KUY	10.9	(9–12)	769.3 ± 300.4	13.5%	45.6%	—	—	—
UGE	11.2	(8–14)	—	13.0%	50.2%	—	—	—
PAB	38.8	(38–39)	730.0 ± 257.6	10.4%	39.1%	116.0 ± 128.2	246.3 ± 286.0	4.3 ± 4.7

Average and range for the number of weaned gorillas per group. Average and standard deviation for the daily travel distances (meters). Proportion of time spent traveling and feeding. Average and standard deviation for the energy intake rates, energy intake per food site, and distance travelled between food sites.

Adult females in the largest group had an average energy intake rate of 116.0 ± 128.2 per minute, which is significantly higher than 81.7 ± 63.9 kJ per minute for two intermediate sized groups (Table 1d). Adult females in the largest group also obtained a significantly higher amount of energy per food site (246.3 ± 286.0 kJ versus 176.8 ± 181.2 kJ for the intermediate sized groups). Adult females in the largest group traveled an average of 4.3 ± 4.7 meters between food sites, which is not significantly different from 4.9 ± 6.8 meters for the intermediate sized groups (Table 1f). The latter two results indicate that the largest group could visit fewer food sites to obtain the same amount of energy as the intermediate size groups, and it did not need to travel farther between those sites, which again suggests that its travel requirements should be lower.

Hypothetically, the higher energy intake rates of the largest group could be distributed across all plant species, or the group could have greater access to more favorable species. To separate those two hypotheses, we ran a post hoc analysis with a subset of the food sites in which a single plant species accounted for at least 80% of the total energy intake. The post hoc analysis was similar to our original model of energy intake rates, except that we added a random effect variable to control for the main plant species of the food site. The category variable for group size was no longer significant in the post hoc analysis (p = 0.22), so we did not find support for the hypothesis that the higher energy intake rates of largest group are distributed across all plant species. Instead, the largest group may have greater access to more favorable species. For example, gorillas had an average energy intake rate of 226.7 ± 196.3 kJ per minute while feeding on *Rubus* spp. versus only 85.1 ± 74.9 for the other plant species. *Rubus* accounted for 30.1% of the total energy intake by the largest group, versus only 6.3% for the other two groups.

## Discussion

Our analyses of daily travel distances and travel times showed a significant quadratic relationship with group size. Foraging efficiency decreased with size among most groups, but then began to increase for the largest group. The largest group also had significantly higher energy intake rates than the intermediate sized groups, which is consistent with the quadratic pattern for our other measures of foraging efficiency. One potential interpretation of these results is that foraging efficiency of most groups was limited by intragroup feeding competition, which the larger group may have been able to mitigate through intergroup competition^23,53^.

Non-linear relationships between foraging efficiency versus group size have also been reported for four groups of woolly monkeys (*Lagothrix lagothricha*) and five groups of savanna baboons (*Papio cynocephalus*), but their patterns were generally in the opposite direction of our study, because foraging efficiency was highest for their intermediate sized groups^32,33^. From a mathematical perspective, the contrasting patterns are represented by the sign of the quadratic term in the statistical models. The significant quadratic term was negative in our analyses of travel times and daily travel distances, but it was positive in four of five multivariate analyses for savanna baboons^32^. A literature review of seventeen studies showed only one significant (positive) quadratic term, and it merely reflected a slight curvature rather than a full U-shaped pattern^7,54^. The rarity of significant quadratic patterns has been attributed to insufficient sample sizes (particularly for smaller groups) as well as weak socioecological influences^32^.

**Figure 3:**
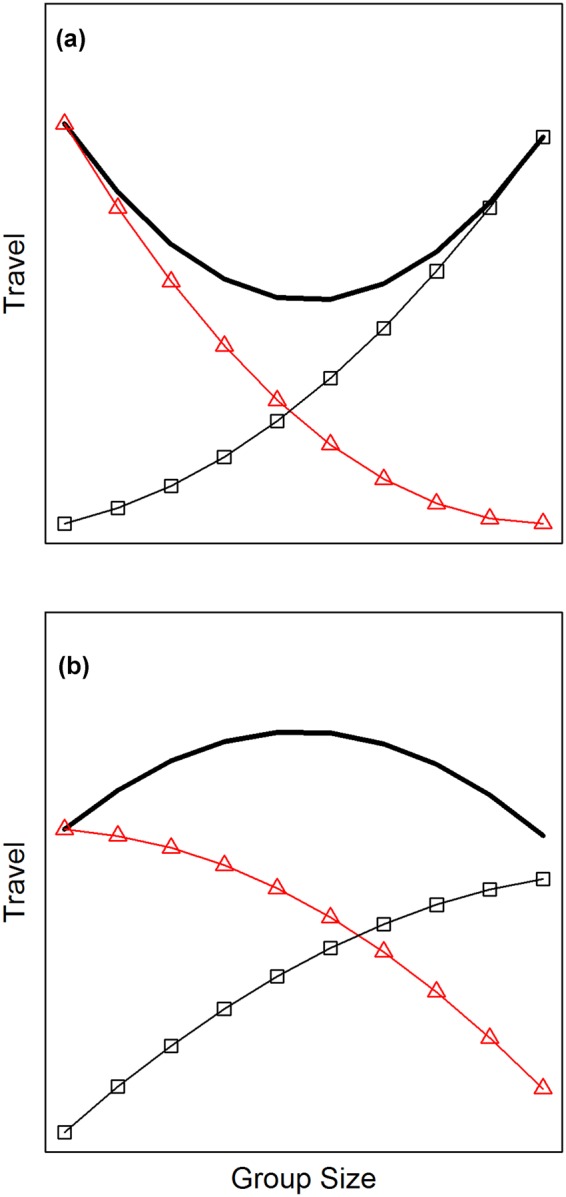
Hypothetical effects of group size on the overall costs of feeding competition (thick line), which equals the combined impact of competition within groups (circles) versus between groups (triangles). The overall costs have a U-shaped pattern if the second derivative is positive for competition both within groups and between groups (**a**). An inverted U-shape arises if the second derivative is negative for both types of competition (**b**). If the two types of competition have opposite curvatures, then the overall pattern will depend on which curvature is stronger (not shown). If both types of competition have no curvature, then the combined effect will also be linear.

From a socioecological perspective, the sign of the quadratic term may reflect the ways that group size affects intragroup feeding competition versus intergroup contest competition (Fig. 3). For example, it could become increasingly difficult for larger groups to avoid the effects of intragroup competition (Fig. 3a), if their ability to increase group spread is limited by the distribution of food^23,35^. Alternatively, groups might not adjust to such competition until it approaches a threshold level (Fig. 3b), if the benefits of subgrouping must be balanced against the risk of predation^55,56^. Similarly, increases in group size might reduce the costs of intergroup contest competition most dramatically for small groups (Fig. 3a), or those cost reductions might not accelerate until groups become larger than average (Fig. 3b). The relationship between group size and intergroup contest competition could even form an inverted U-shaped pattern, with the greatest costs incurred by mid-sized groups^57^. Further study is needed to disentangle these nuances of each type of competition^5,29^.

**Figure 4:**
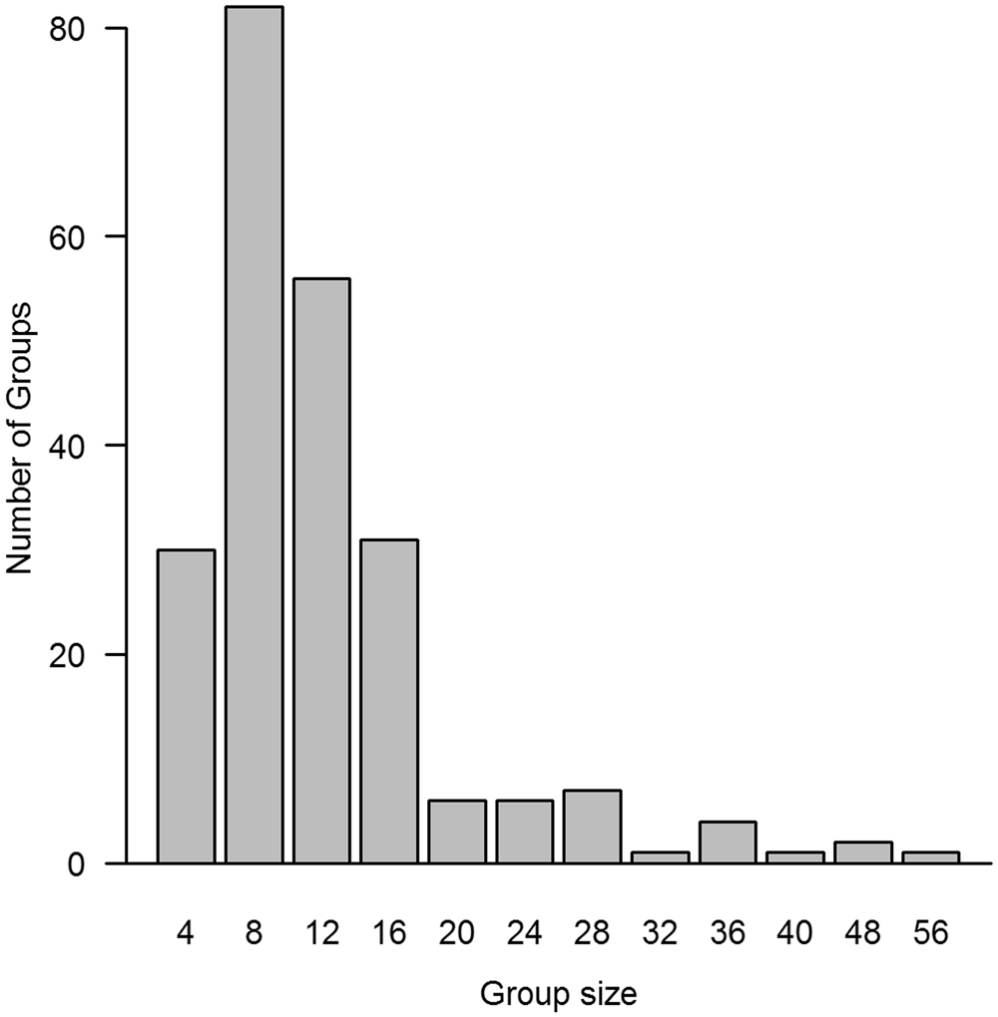
Group size distribution of the Virunga mountain gorillas. The y-axis indicates the number of groups that were recorded within each size category during eight censuses of the entire population from 1971–2010^74,88–95^. The numbers along the x-axis represent the largest group size that was included in each category.

If foraging efficiency is the primary influence on individual fitness and behavior, then the aforementioned U-shaped patterns for woolly monkeys and savanna baboons should lead to narrower group size distributions, which would be analogous to stabilizing selection for genetic traits^58,59^. Small groups would benefit from getting larger; and large groups would benefit from getting smaller. In contrast, the inverted-U pattern in this study should lead to a broader (and ultimately bimodal) group size distribution, which would be analogous to disruptive stabilization^60,61^. A comparative study of group size distributions has not been reported for primates, so it is unclear whether the variance is especially broad for Virunga mountain gorillas (Fig. 4). Their group size distribution is not bimodal, however, which suggests that our results are not typical of the overall population, or that foraging efficiency is less influential than other factors such as the quality and quantity of adult males^44,62–64^. The breadth of group size distributions can also depend on variability in ecological conditions, as well as the flexibility of individuals to adjust to such conditions, particularly via patterns of dispersal and group fissioning^32,58^.

Previous studies of this population did not find the inverted-U pattern, perhaps because they did not encompass the size of our largest group^35,45^. Nonetheless, over the past 50 years of research and ecotourism in the Virunga Volcano region, seven of the 39 (18%) habituated groups have contained at least 25 gorillas, which is more than two standard deviations beyond the average value for the other habituated groups^65^. Thus the tail in Fig. 4 does not represent an isolated incident, suggesting that large groups may be beneficial but limited in occurrence by other factors such as the ability of a single male to attract and retain a large number of females (mate competition). Group size was not significantly correlated with female reproductive success in a study that included five of those seven large groups, which supports our finding that such groups are not constrained by foraging efficiency^44^. The probability of female emigration was significantly lower in larger groups, which may indicate that females prefer to reproduce in such groups^64^. The latter result was merely interpreted as further evidence that intragroup feeding competition is minimal, yet it could also suggest that larger groups have an advantage in intergroup competition.

The largest group had only two known encounters with other groups throughout the study, so any advantage in intergroup contest competition did not arise by routinely displacing its competitors from patches of food. Instead, the largest group had far less home range overlap than the other study groups (~20% versus 90%), which is more consistent with an avoidance mechanism of intergroup contest competition^52,66^. Among the other groups, greater home range overlap likely caused more BGS, which may have exacerbated WGS. The home range comparisons do not include any overlap with non-study groups, but the largest group had no known encounters with those groups during this study, so its overlap with their home ranges may have been minor. Other large groups have also had low home range overlap and/or low rates of intergroup encounters, but statistical correlations have not been reported between those parameters and group size^52,67^. Groups may avoid each other by travelling farther after encounters, by using long-distance signaling mechanisms (e.g. chest beats), and by moving away from areas where foraging has already occurred^68–70^.

The avoidance mechanism is probably driven by male mating competition, which predominates the behavioral interactions when mountain gorilla groups meet^42,47,70^. Such avoidance may help males to retain their females, who disperse only during encounters with other adult males^43,71^. In some cases, a large group may even avoid smaller groups, because it has more females at stake during such encounters^35,45^. Indeed, the home range of the largest group gradually shifted farther from the other study groups between 2005–2011, which may indicate that it was avoiding an area where group density was increasing^52^. The smaller groups have not expanded into the same area as the largest group, however, perhaps because they wanted to avoid intergroup competition with it. Thus, the largest group generally had the lowest degree of home range overlap throughout the ten years prior to our study^52^. Analysis that would reveal if larger or smaller groups are avoiding each other in this population remains to be done. Just as dominance hierarchies among individuals are used to evaluate intragroup contest competition, evidence of a dominance hierarchy among groups could help to clarify the effects of intergroup contest competition on ranging patterns, but previous studies of mountain gorillas do not indicate an obvious winner of most encounters^42,47^.

Interestingly, time spent feeding showed a significant negative correlation with group size. Similarly, the recent study of baboons showed a linear relationship between group size and time spent foraging (feeding and moving), despite a quadratic relationship for other measures of foraging efficiency^32^. Again, however, their results were in the opposite direction of ours, because the foraging time of baboons increased with group size^32^. Their positive correlation could indicate an influence of group size on intragroup feeding competition, whereas our negative correlation is more consistent with theoretical predictions for intergroup contest competition^7,8^. The feeding time of mountain gorillas seems to be more sensitive to differences in energy intake rates than differences in the energy requirements from travel^36,41,72^. If so, then our negative correlation could be interpreted to suggest that larger groups had greater access to areas that facilitate higher energy intake rates.

The higher energy intake rate of the largest group arose mainly from the consumption of *Rubus* plants, which are especially concentrated on the western edge of our study site. Thus, for the largest group, shifting its range slightly to the west, perhaps to avoid an area of greatly increased group density, also exposed it to high quality habitat. In which case, its higher energy intake rate could be a result of being in the right place at the right time rather than outcompeting smaller groups. *Rubus* has increased in biomass over the last 20 years (from being present in 1% to 26% of sampled plots), which may also help to explain why our results do not match previous studies^35,45,73^. *Rubus* does not seem to entirely explain our quadratic relationships, however, because the results for daily travel distance remained significant even when we excluded the largest group.

The population of the Virunga mountain gorillas has doubled in size over the past few decades, which provides an opportunity to evaluate the impact of between-group scramble competition upon their foraging patterns and food availability^74^. The average daily travel distance in this study was approximately 25–40% longer than one group in the late 1970s, and the proportion of time spent traveling was 50–70% greater than previous data from four groups^35,39,75^. Home range sizes have remained unchanged^45,52^. The biomass density declined by more than 50% for two of their most frequently consumed foods, but it increased for three of their other top-five foods^73^. Some of these apparent changes could be due to differences in methodology, and collectively the results do not indicate an immediate threat to continued population growth, but they highlight areas that warrant continued monitoring for this critically endangered species^52,65,73^. The changes in population density and food density could also intensify both intragroup feeding competition and intergroup contest competition, thereby contributing to the quadratic relationships that were detected in this study.

## Methods

### Study population

We studied nine groups of habituated mountain gorillas that are monitored by the Dian Fossey Gorilla Fund’s Karisoke Research Center in the Volcanoes National Park of Rwanda (Table 2). The Virunga Volcano region contains a range of habitats that vary along an altitudinal gradient from the bamboo zone below 2800 meters to afro-montane vegetation above 3600 meters^36,39,45,73^. Between those two extremes, the additional habitats are the mixed species montane forest with an understory of *Mimulopsis* spp. at 2500–2700 m, the Hagenia- Hypericum woodland found in the saddle between two volcanoes at 2800–3300 m, the Hypericum woodland (a.k.a. brush ridge) on the volcano slopes at 3000–3300 m, the herbaceous vegetation zone containing dense tall herbs with no tree cover at 2800–3300 m, and the subalpine zone containing stands of *Lobelia stuhlmannii* and thickets of *Rubus* spp. at 3300–3600 m. The Virunga mountain gorillas primarily feed on herbaceous vegetation that is available year-round, except for bamboo shoots which were consumed from October through December during this study^76–78^.

### Data collection

We measured the daily travel distance of five groups from September 2009 to September 2010 (n = 7.5 ± 1.8 measurements per group-month, Table 2). Each adult gorilla makes a nest at night and the group creates a trail of trampled vegetation throughout the day. We followed the main trail of the group, and took readings every 30 seconds using the “track log” function of a GPS (accurate to within ca. five meters). The daily travel distance was calculated as the sum of distances between each set of coordinates in ArcGIS®. Some nest-to-nest distances covered several altitudinal zones and were corrected for terrain using a Digital Elevation Model with 50 m contours (courtesy of M. Gray). Daily travel may be influenced by attempts to avoid other groups and/or solitary males^68^, so we excluded eight days when the focal group was involved in an intergroup encounter.

We measured the activity budgets of 79 gorillas in nine groups from October 2009 through December 2010. During 50 minute focal sessions of each individual, the protocol involved taking an instantaneous scan every 10 minutes to record the activity (feeding, traveling, resting, grooming, or playing). The analyses were limited to gorillas who were at least eight years old, which is when females are typically considered adults. Males are not considered adults until age twelve, but by age eight they are already as large as adult females, and activity budgets are similar among age-sex classifications^39^. As required by the Rwanda Development Board, observations of the gorillas were limited to four hours per day to minimize anthropogenic disturbance.

Energy intake data was taken from Grueter (2016), which analyzed food sites for 23 adult females in three groups from October 2009 through December 2010. The energy intake rate for each food site equaled the total energy intake, divided by the food site residence time (FSRT). The FSRT was defined as the elapsed time from when a female settled at a food site and commenced eating, until she stopped eating and/or moved more than one meter^72,79^. The energy intake for each food site equaled the number of food “units” consumed during the FSRT (e.g., one leaf or stem of a specific plant species), multiplied by the average energy content for each type of unit^80,81^. The energy content for 33 types of food units from 25 species was calculated from nutritional analyses of crude protein, lipids, neutral detergent fiber, and total ash; as conducted by the Leibniz Institute for Zoo and Wildlife Research in Berlin. For the 23 adult females in the three study groups, we collected 657 hours of data with the energy intake protocol and 931 focal hours with the activity budget protocol, which represent 28.5 and 39.9 hours per female, respectively.

### Statistical analyses

To analyze the daily travel distances, we ran a linear mixed model with one data point for each day that each group was measured (n = 448 data points). The response variable was the distance that the group traveled (meters). The predictor variables were the group size (the number of weaned individuals) and group size squared. A positive term for group size squared would represent a U-shaped pattern, with the shortest daily travel distances occurring at intermediate group sizes, and longer travel distances for the smallest and largest groups. A negative term for group size squared would represent an inverted U-shaped pattern, with the longest daily travel distances occurring at intermediate group sizes, and shorter travel distances for the smallest and largest groups. We included the daily rainfall as a control variable because the gorillas typically stop travelling when it is raining^35,82^. We also added a control variable to account for potential temporal autocorrelation among the data points within each group^83^. The random effect variables were the group ID and the habitat ID. The latter random effect variable may help to control for spatial variations in food density^11,35,39^. To account for the seasonality of bamboo shoots, the random effect variable of habitat ID included one category for its habitat during the bamboo season, and another category for its habitat during the rest of the year.

To examine the proportion of time that gorillas spent traveling, we ran a linear mixed model with one data point for each habitat that each focal gorilla used on each day (n = 3866 data points). For example, if the group used two habitats during focal observations of one gorilla, and only one habitat during the focal observations of another gorilla, then the model would have three data points for that day. For each data point, the response variable equaled the duration of time that the gorilla spent traveling, with an offset control variable for the total duration of their focal time (traveling plus all other activities). Our approach is similar to using proportion of time spent traveling as the response variable, but the control variable helps to avoid excessive influence from data points that are based on brief observations^84^. The predictor variables were the group size and group size squared. The control variables were the daily rainfall and the term for temporal autocorrelation. The random effect variables were the gorilla ID, group ID, and the habitat ID. We ran a similar model to examine the proportion of time that gorillas spent feeding.

To analyze the energy intake rates, we ran a linear mixed model with one data point for each food site (n = 3342 data points). The response variable was the total energy intake at the food site (kJ), with an offset control variable for the time spent feeding at the site (minutes). The number of groups was insufficient to look for nonlinear effects of group size (or even a strong test of linear effects), so the predictor variable merely tested whether the largest group (PAB) was significantly different from two intermediate sized groups (NTA and BWE). The random effect variables were the gorilla ID, group ID, and the habitat ID; and we added a control variable for temporal autocorrelation. We ran a similar model to analyze the energy intake per food site. The response variable again was the total energy intake at the food site (kJ), but we omitted the control variable for the time spent feeding at the site. We ran a third model in which the response variable was the distance travelled between consecutive food sites. For all three models, the random effect for gorilla ID can help to control for female rank, which did not show a significant effect on these response variables in the previous study of this data^41^.

All linear mixed models were run with a Gaussian error structure and identity link while using the “lmer” function of the “lme4” package in R^85^. We used log or exponential transformations of the response variables as needed to obtain more normally distributed residuals. The predictor variables for group size were log transformed to provide a more uniform distribution of values, and then were also standardized so they each had a mean value of “0” and a standard deviation of “1”^86^. We obtained p-values for each predictor variable by using the “drop1” function, which performs likelihood ratio tests to compare the full model versus a set of reduced models that exclude one predictor at a time^87^.

## Data Availability

The datasets generated during and analyzed during the current study are available from the corresponding author on reasonable request.
